# Dimensional and Geometrical Quality Enhancement in Additively Manufactured Parts: Systematic Framework and A Case Study

**DOI:** 10.3390/ma12233937

**Published:** 2019-11-28

**Authors:** Natalia Beltrán, David Blanco, Braulio José Álvarez, Álvaro Noriega, Pedro Fernández

**Affiliations:** Department of Construction and Manufacturing Engineering, University of Oviedo, Pedro Puig Adam St., E.D.O.5, 33203 Gijon, Asturias, Spain; nataliabeltran@uniovi.es (N.B.); braulio@uniovi.es (B.J.Á.); noriegaalvaro@uniovi.es (Á.N.); pedrofa@uniovi.es (P.F.)

**Keywords:** additive manufacturing, quality enhancement, process parameters, design optimization

## Abstract

In order to compete with traditional manufacturing processes, Additive Manufacturing (AM) should be capable of producing medium to large batches at industrial-degree quality and competitive cost-per-unit. This paper proposes a systematic framework approach to the problem of fulfilling dimensional and geometric requirements for medium batch sizes of AM parts, which has been structured as a three-step optimization methodology. Firstly, specific work characteristics are analyzed so that information is arranged according to an Operation Space (factors that could have an influence upon quality) and a Verification Space (formed by quality indicators and requirements). Standard process configuration leads to characterization of the standard achievable quality. Secondly, controllable factors are analyzed to determine their relative influence upon quality indicators and the optimal process configuration. Thirdly, optimization of part dimensional and/or geometric definition at the design level is performed in order to improve part quality and meet quality requirements. To evaluate the usefulness of the proposed framework under quasi-industrial condition, a case study is presented here which is focused on the dimensional and geometric optimization of surgical-steel tibia resection guides manufactured by Laser-Power Bed Fusion (L-PBF). The results show that the proposed approach allows for part quality improvement to a degree that matches the initial requirements.

## 1. Introduction

Additive Manufacturing (AM) is defined as “the process of joining materials to make objects from 3D model data, usually layer upon layer, as opposed to subtractive manufacturing fabrication methodologies” according to ISO 17296-1 [1] and ASTM 2792-12 [2]. This definition encompasses a wide variety of processes used to manufacture three-dimensional objects by means of vertical stacking of bi-dimensional layers. Most of the technologies involved have gained maturity in recent years. This has allowed AM applications to evolve from prototype manufacturing to small-batch-size production. Nevertheless, according to Gartner´s hype cycle, consistent adoption of AM in manufacturing operations will still take 5 to 10 years of development [3]. There are many factors that influence AM’s difficulties to match the requirements of medium and high batch-size production. Some are related to working volumes and production rates of machines at the current state of development. Others reflect the difficulty of producing parts with similar mechanical behavior to those obtained by traditional manufacturing processes. Finally, dimensional and geometric quality deficiencies of AM parts have also been highlighted as common disadvantages [4,5], which explains their relevance as research subjects during the last decade [6,7,8,9,10]. Quality improvement is a *sine qua non* condition for the generalized industrial adoption of AM processes, since cost-per-unit reduction would not be enough by itself. Research in the field of dimensional and/or geometric quality improvement of AM parts could be grouped according to three different approaches: error analysis, error prevention and error correction.

Error analysis pays attention to the influence of process parameters on the dimensional or geometrical accuracy of parts. The usual error analysis approach involves comparing the theoretical values of the design parameters and their correspondent values measured upon the manufactured part [11,12,13]. Consequently, these works provide useful information regarding the expected results of an AM process in terms of quality and accuracy. Research under this approach shows a wide variety of factors influencing the accuracy of AM parts. This is related to the variety of physical principles and implementations available, which lead to specific research solutions for each individual case. These studies also show a lack of uniformity regarding the indicators used to compare the results of modifying process configuration. Researchers sometimes use indicators with no real meaning from an industrial point of view [11,13]. Tests are also frequently carried out upon test specimens and geometries designed *ad hoc* [11,12].

Approaches based on error prevention aim to establish the optimal process configuration to manufacture parts with the highest achievable quality. This goal is commonly based on the analysis of possible error sources and their relative influence upon quality, so that their effects could be minimized by means of parameters adjustment. Once again, the variety of physical principles and configuration parameters used in different AM processes hinders the adoption of a unified methodology for error prevention. Deposition speed or scanning energy are among the factors considered under this approach, but factors related to practical decisions like part location and orientation [6,14] are also used. This last category aims to provide useful recommendations on the best way to place the parts on the working volume. Finally, internal part model parameters, like raster angle or layer thickness [15], are also analyzed. This approach could explore the possibilities of a given technology to improve quality by acting upon influence factors. Nevertheless, this could also be its main limitation, since once the optimal configuration has been determined and errors still exceed tolerance requirements, there is no margin for further improvement.

Finally, error correction approaches act upon accuracy by working on the strategies used to convert 3D geometry into a series of flat layers (slicing), on the generation of material deposition paths and tool trajectories, or directly upon the CAD model. Therefore, this group is formed by those approaches that intend to overpass the limitations derived from the combination of process, technology and geometry to improve part dimensional or geometrical quality. This global objective could be achieved by different solutions. Some works just compensate deviations from the theoretical values [16]; others aim to compensate mechanical errors in the machine [17]; some works elaborate complex models to compensate the influence of different parameters upon the overall quality [18]. In recent years, there has been a tendency to apply machine-learning methods to provide error correction in AM [10,19,20]. Some works focus on compensating in-plane shape deformation [20,21]. Other approaches work with the 3D geometry, mainly compensating thermal deformations that have previously been modelled via finite elements (FE) simulation [22] or by means of virtual manufacturing models [23,24]. These types of works build “predictive” mathematical models that could be based on experimental data [10,20] or build from theoretical models [21,23]. Although Artificial Neural Networks (ANN) are frequently used for building predictive models [10,21,22], alternative mathematical modelling is also used for this purpose (e.g., Gaussian process multi-task learning [20] or particle swarm optimization [25]. The information provided by predictive models could be later used to change input parameters in order to fulfil tolerances. 

In sum, the objective of improving dimensional and geometric quality in AM parts is frequently addressed without a recognizable methodology or a standardized procedure, mainly due to the variety of processes and influence factors. This situation is even more pronounced since most of the research has been carried upon “laboratory specimens”, neglecting the relevance of industrial tolerances or the problems derived from medium to large batch productions. Additionally, most of the research has been carried out upon parts designed *ad hoc*, with different levels of complexity, that greatly differ between studies. These artefacts frequently consist of a collection of basic geometries (planes, cylinders, spheres) arranged in one unique part. There are also examples of dimensional quality comparison between biological-type parts (usually bones) in the fields of surgical reconstruction. Organizations like the American National Institute of Standards and Technology (NIST) have even proposed their own round-robin artefacts for establishing repeatability or reproducibility values for a particular AM technology or machine [26]. Accordingly, there are huge differences between quality indicators: some could be considered as artificial indicators, since they are calculated through complex mathematical expressions that ponder a series of individual parameters; others are usually referred to as “volumetric errors” although they lack a standardized physical meaning. Both types of indicators are useful to provide an impression of the overall manufacturing accuracy or for comparison between different process configurations, machines or technologies. Nevertheless, they tend to ignore the fact that dimensional tolerances in industry are limited to Features of Size (FoS) [27], as they are related to fit purposes. Finally, another frequent problem is that error is sometimes assumed to be linear, whereas, in AM processes, there are many factors that could have a non-linear influence upon error, like volumetric shrinkage of thermoplastic in Material Extrusion (ME) processes. 

In the present work, a systematic framework for the minimization of dimensional and geometrical errors and tolerance fulfilment in AM parts is presented. This methodology has been specially developed to be applied upon FoS and medium to large production batches. In the following sections, a description of the framework will be presented. The systematic approach is structured in three consecutive steps: Work Analysis, Process Optimization and Design Optimization. The methodology has been evaluated using a case study under quasi-industrial conditions, which is the dimensional and geometric optimization of surgical-steel tibia resection guides manufactured by L-PBF.

## 2. Systematic Framework Description

The proposed framework has been designed to be used when production of a given part simultaneously fulfils two conditions:

- At least one of its features is a Feature of Size. Consequently, part geometry must include at least one cylinder or two parallel opposite planes [27] affected by a dimensional tolerance;
- Batch size and part-added value justify a proportional investment of test specimens and optimization effort.

**Figure 1:**
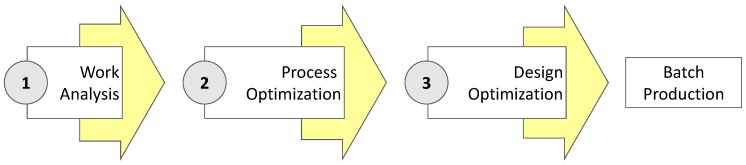
Steps of proposed approach.

Therefore, parts with features used for fitting purposes would be candidates for optimization via the proposed approach, whereas parts with features affected only by general tolerances would not be worth of such optimization efforts. Similarly, small batch sizes would not justify the effort of a systematic optimization. In these cases, alternative improvement strategies (e.g., trial-and-error) should be considered. Three consecutive stages have been proposed for the optimization: Work Analysis, Process Optimization and Design Optimization (Figure 1).

### 2.1. Work Analysis

**Figure 2:**
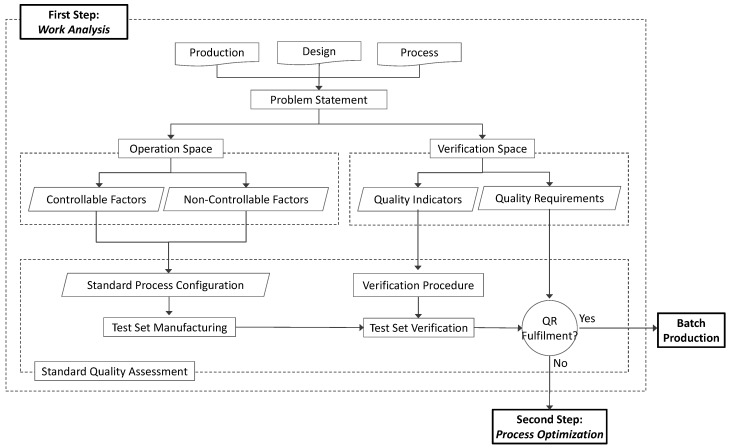
Work Analysis workflow.

Firstly, a preliminary analysis of the work to be done would be carried out, with the objective of achieving a full description of the problem and evaluating lack-of-quality issues. This implies collecting all the information regarding part, process, production and equipment to elaborate an initial problem statement, defining an operational space and performing an initial quality characterization (Figure 2). Input information should be collected and structured according to three categories—production requirements, design specifications and process characteristics:

- Production requirements encompass information about expected production rates, batch size, maximum profitable manufacturing cost-per-unit and all additional requirements—like expected strength or hardness—that would not be related to geometrical requirements;
- Design specifications would comprise the geometrical information of the 3D CAD file, along with dimensional and geometric tolerances requirements. Information about shapes and dimensions should be taken into account even if they are not directly affected by tolerances. Part material should also be included in this category;
- Process characteristics should include all data regarding AM processes and equipment within the scope of the problem. Depending on each particular situation, process and/or machine could be previously set or included in the problem (which implies that process would be considered as an additional factor). At the process level, staff in charge of the optimization must analyze the information about the fundamentals of the process, including physical principle, range of materials or common manufacturing defects. At the equipment level, attention should be paid to the characteristics of machines, like workspace dimensions, axes speed, operation limits, or appropriate ranges for configuration parameters (layer thickness, building speed, etc.). Batch size and part-added value justify a proportional investment on test specimens and optimization effort.

Once all the relevant information has been collected, a statement of the problem should be performed. The objective of this task is to define both an Operation Space and a Verification Space.

- The Operation Space would consist of all those factors that could have an influence upon part quality. This means that every single factor whose modification would presumably affect, to a certain degree, the quality of the part, must be included in the Operation Space. Factors could be subject to modification (controllable factors) or not (non-controllable factors). Controllable factors are those that can be modified according to production decisions. This category includes discrete factors (e.g., selecting “glossy” or “matte” finishing in a Material Jetting process) and continuous factors (e.g., nozzle temperature in ME of thermoplastics) that could adopt many different values within a certain range. On the other hand, non-controllable factors are those that, having an influence upon part quality, should not be modified. This category would include design decisions of requirements (shape, dimensions, material, etc.) that have been set during the design stage. It also should include production decisions (batch size, process, production machine, etc.) that are not subjected to possible modifications. Process parameters could be also considered non-controllable factors when their values have been set according to material or machine supplier recommendations, workshop procedures or workforce experience. Categorization of influence factors into non-controllable and controllable groups is a key task. Most factors undoubtedly belong to one of these groups, but special attention should be paid to those factors that do not have a significant influence upon quality according to previous know-how, since there is a risk of neglecting their influence upon a particular part or feature, despite their actual significance;
- The Verification Space would be formed by Quality Indicators (QI) and Quality Requirements (QR). QI would be used for evaluating the degree of compliance of the tolerances imposed during the design stage. They would usually match FoS quality requirements, like dimensions (diameter of a cylindrical feature or distance between parallel flat surfaces) or geometrical deviation of controlled features (flatness, parallelism, cylindricity or concentricity). Nevertheless, QI could also be defined as relative differences between those parameters and their optimal values (e.g., the difference between the measured diameter and the middle value of the tolerance interval). During the definition of QI, it would also be necessary to define the verification procedure. This means that an inspection plan should be elaborated, including the materials and methods used for verifying each part and calculating actual values for each QI. Finally, QR are defined as the range of acceptable values that each single QI should adopt to enable batch production.

Once information has been structured into the Operating Space and the Verification Space, the objective of this first step is to determine if a standard process configuration could ensure the fulfilment of QR. In order to check this condition, a test set must be manufactured and verified. This implies that staff in charge of improving part quality must decide which process configuration should be used, by setting all controllable factors. This task should be performed taking into account previous experiences, good practices and workforce know-how.

The size of the test set must be determined in order to properly check QR fulfilment and, simultaneously, minimize the number of test specimens to be manufactured. Robustness of this task will increase with the number of replicas, whereas test size is conditioned by experimental cost. Nevertheless, a minimum of two building trays for each particular manufacturing configuration should be demanded, in order to contemplate experimental error. In accordance, calculation of QI values should also be done by means of arithmetic average values of repeated measurements. Staff are encouraged to consult the available literature regarding Design of Experiments (DOE) and Quality Assessment [28].

Manufactured test specimens would then be measured by means of the verification procedure, so that measured values for QI would be calculated and compared with QR. If the results indicate that those requirements would be fulfilled for an acceptable number of parts (defined as the percentage of valid parts per total production), then parts would be considered suitable for batch production and the procedure would finish. If requirements are not appropriately fulfilled, then the strategy continues through its second step. An intermediate situation could also occur when some of the QR are fulfilled but not all of them. In this case, the efforts during Process Optimization should be focused on those requirements that have not been properly fulfilled.

### 2.2. Process Optimization

**Figure 3:**
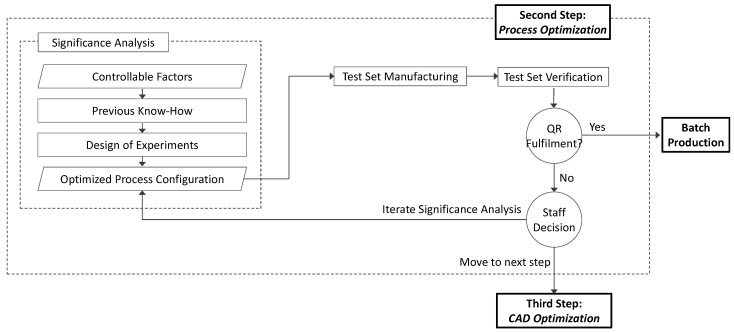
Process Optimization workflow.

Process Optimization (Figure 3) determines if quality requirements could be fulfilled by just acting upon controllable factors. The complexity of testing the significance on quality of variations in Controllable Factors sharply increases with their number. Checking their influence could be simple when few factors have to be considered, but turns to be extremely complex when the number of factors that should be tested increases. To minimize this problem, factors could be ranked according to their level of significance by means of statistical tools, like Design of Experiments (DOE). This Significance Analysis would be performed as an iterative task to reduce experimental effort, since considering all possible Controllable Factors for DOE could be an inefficient approach when some initial restrictions based on previous know-how have been incorporated into the analysis. Consequently, an initial appreciation of each factor significance could be established based on research papers results, reference books, workforce know-how, etc. This approach would help to fix some factors and reduce experimental effort. 

For instance, it is widely accepted that layer thickness affects geometric quality in ME, since coarse layers increase the staircase effect of sloped surfaces. Consequently, although layer thickness is usually a controllable factor and could be considered during Process Optimization, it can be assumed that increasing layer thickness would not improve geometric quality, and thus this factor should not be included in the optimization step. 

Once the number of factors has been initially reduced by means of previous know-how, further decisions should be sustained by experimental testing and supported by statistical analysis. Experimental designs could demand huge experimental effort when a high number of factors are considered. In these cases, the use of fractional factorial designs is widely recommended. Fractional designs would allow for reducing the experimental error by minimizing the number of experiments (and, consequently, the cost of manufacturing and measuring test specimens) by running a fraction of a full factorial design. This approach has the disadvantage of confounding effects of higher-order interaction, but it will be useful to characterize main effects and low-order interactions at a reduced experimental cost. Accordingly, an analysis of variance could provide an ordered list of factors, reflecting their relative influence upon each QI variance. Factors that show no influence upon QI should no longer be considered for Process Optimization. On the other hand, those factors that show a significant influence upon QI results should be ranked according to their relative importance. This procedure could lead directly to an optimized process configuration if only categorical factors (fan on/fan off) have been considered. Nevertheless, if continuous and discrete factors have been included in the DoE, further research could be demanded. In any case, analysis of variance would provide a regression equation that models how QI behaves according to changes in the influence factors. This equation should be used to optimize a particular QI, whereas multiple response regression optimization methods could be used for simultaneous optimization of multiple Qls.

This optimization effort should lead to a newly optimized process configuration and, once this initial optimization has been established, a new test set should be manufactured in order to check if the QR are fulfilled. A positive result would lead to starting batch production under the optimized configuration, whereas a negative result would lead to revising the significance analysis. If no completely positive result could be achieved, staff should take the decision to finish this step and move onto the Design Optimization step.

### 2.3. Design Optimization

Part manufacturing after Process Optimization may still produce features that do not fulfil expected QR. Consequently, part errors (deviations between QI values and optimal QR) should be mathematically modeled and design parameters that could have an influence on the results must be identified. It has to be noted that, in this methodology, design factors are limited to those that could be modified at the CAD definition step. This means that inner-part characteristics like layer thickness or wall thickness are considered process factors, since they are defined at the Computer-Aided Manufacturing (CAM) step. 

There are clear differences regarding the level of complexity of error modelling for the non-fulfilment of dimensional QR and geometrical QR:

- Dimensional non-fulfilments could be corrected if the observed variability of QI is comparatively lower than the range of acceptable values defined by each correspondent QR. In these cases, Design Optimization searches for a model where the values of design parameters related to QI could be modified to improve quality. This means that optimization could be conducted without modifying the initial parameterization of the part, since only the values of existing parameters would be modified according to the correspondent inverse model;
- Geometrical non-fulfilments, on the other hand, demand the change of part parameterization at the design stage, which could be a more complex task. In this situation, a model of geometric distortion would be necessary. The ideal situation at this point is that analysis of part geometric distortion leads to a recognizable pattern that could be easily parameterized—e.g., if a theoretically cylindrical feature resembles an elliptic cylinder once manufactured, the geometric distortion model should determine the orientation of the major and minor axis, and the correspondent equation coefficients and re-parameterization turns, to be almost direct. Nevertheless, in other situations, modelling the deformed geometry would require methods of higher complexity. E.g., if the deformation does not resemble any common primitive, the theoretical cylinder could be modelled by means of a free-form adjustment based on Non-Uniform Rational Basis Splines (NURBS). Research efforts should be conducted in order to select the most appropriate fitting model, minimizing as much as possible the model complexity and taking into account each particular CAD suite capability for manipulating different types of parameterization. In both cases, original parameterization should be substituted with a new parameterization that follows actual manufactured geometries. Once this objective is achieved, the problem is similar to that described for dimensional non-fulfillments (defining a predictive model and an inverse model) with the difference that, in this case, alternative design parameters are used.

Additionally, it must be taken into account that, although geometrical re-parameterization could influence dimensional results, it is possible that dimensional optimization would not significantly influence geometrical QR fulfilment. This fact leads to the proposal that geometrical optimization, if necessary, should be carried out before dealing with dimensional optimization. 

Once an adequate parameterization has been defined, it would be used to predict the most probable value that each QI would adopt in the final part as a function of controllable factors and design factors. Consequently, a new set of test specimens that include variations in the values of those design parameters that influence QR fulfillment would be manufactured and measured, and QI measures would be used to elaborate a “predictive” model. 

There are many mathematical models that could be used at this step, ranging from simple linear regression to complex computing systems (response surfaces, artificial neural networks, etc.). The staff in charge of optimization should evaluate, in each case, which would be the best option for building a predictive model, pondering the required experimental effort and model complexity. 

**Figure 4:**
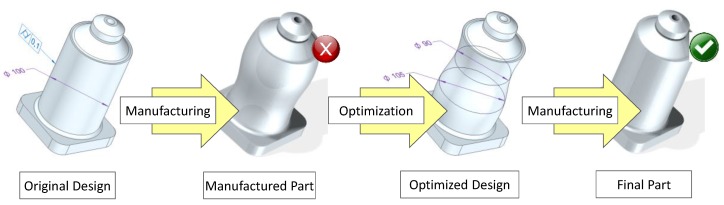
Design Optimization strategy.

Once the predictive model has been made available, the objective is to define a new mathematical model that answers the inverse question: what should the proper values of design parameters be to obtain a QI as close as possible to the optimal QR? This new model should be known as an “inverse” model. The inverse model would provide new values for design parameters, so that a newly optimized design could be obtained. Ideally, new parts manufactured with this optimized design would be closer to the design theoretical objective, fulfilling the desired QR. Figure 4 contains a graphical explanation of this strategy.

**Figure 5:**
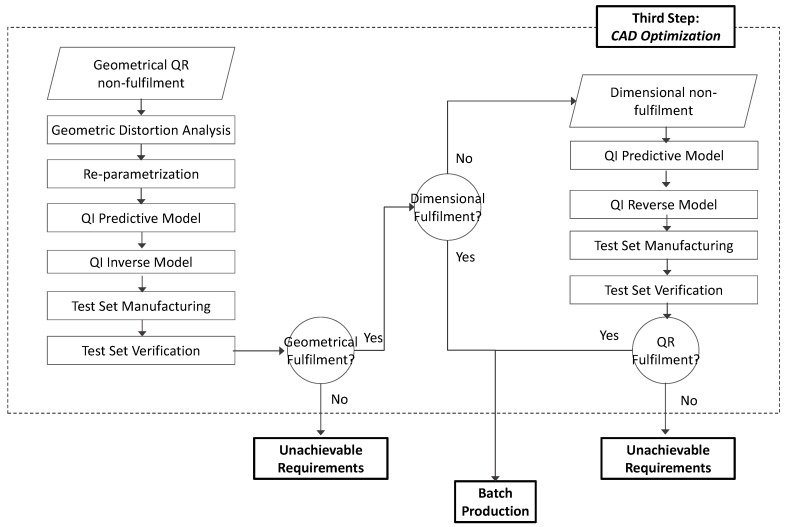
Design Optimization workflow.

The proposed workflow for the Design Optimization step is provided in Figure 5.

### 2.4. Practical Recommendations

Once the different steps of the methodology have been described, there are some practical recommendations that should be taken into account:

- Optimization steps should be carried out using the minimal number of experiments and also minimizing the number of test specimens that would be manufactured;
- Subjective decisions regarding process configuration should be avoided whenever possible;
- Several tasks could have an iterative application when results suggest that additional research could be worthwhile;
- Factors categorized as non-controllable by following previous know-how or good-practices recommendations could have a more significant influence than previously thought for certain geometries. Accordingly, if QI variance could not be put under control by means of controllable-factor adjustment, such categorizations must be carefully revised.

To evaluate the usefulness of the proposed framework, a case study is presented in the next section.

## 3. Case Study: Optimization of Surgical-Steel Tibia Resection Guides

**Figure 6:**
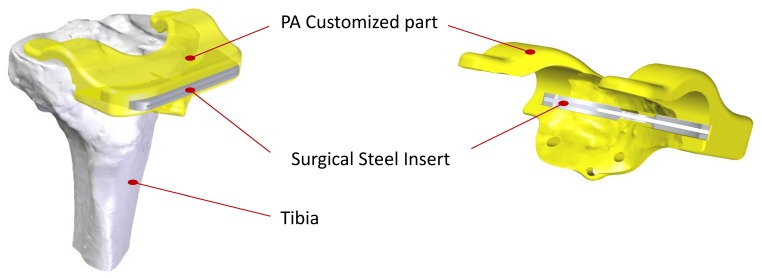
Parts of a bi-component tibia resection guide.

Evaluation case studies must fulfil two conditions: the design must contemplate at least one FoS affected by a dimensional tolerance, and optimization effort must be in accordance with potential batch size. Under both premises, several alternatives were considered until a surgical-steel tibia resection guide was finally selected. This part is a metallic insert used for the guidance of resection instruments during knee arthroplasty, a surgical procedure that is carried out approximately 600,000 times a year in Europe according to EUROSTAT statistical reports [29]. Among different alternatives for resection tool guidance, the one considered here (Figure 6) is a bi-component design, consisting of a polyamide customized alignment part (single-use) and a surgical steel insert (multiple-uses).

From a functional point of view, metallic inserts must fit into the PA part and become a single element (no relative movement allowed). At the same time, the theoretical resection plane should be accurately oriented with respect to the alignment features. Guidance is achieved by means of a deep and narrow through slot, defined by two parallel opposite flat surfaces and two slightly tapered lateral sides, resembling an arrow slit, to facilitate instrument handling. This geometry can be obtained through traditional manufacturing processes in two stages. Firstly, basic geometry could be manufactured directly by milling a metal plate or a casting preform; secondly, although external geometry could be completed by milling, the slot is so narrow and deep that it should be manufactured by means of Wire EDM (Electro Discharge Machining) or Sinker EDM. The combined manufacturing costs of these processes could range from 100 to 300 € per unit according to TEKNOS experts. Nevertheless, such geometry could be also manufactured by means of metal AM processes at a competitive cost.

In following sections, the consecutive steps of the proposed methodology applied to such geometry will be explained and discussed.

### 3.1. Step 1: Work Analysis

**Figure 7:**
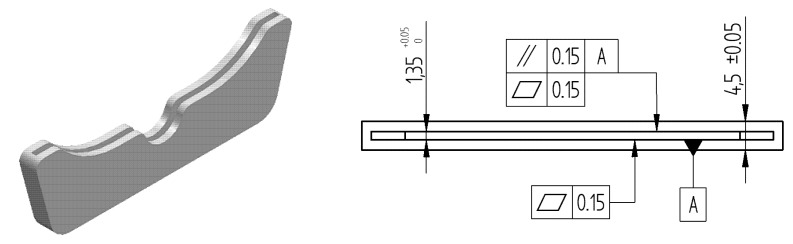
Part design (**a**) Perspective view; (**b**) Main tolerances in mm.

Work analysis starts by gathering together all available information about production requirements, design specifications and process characteristics. Firstly, inserts must be manufactured in a material suitable for biomedical applications, like surgical stainless steel or titanium, by means of a process capable of achieving good quality. This condition led to selecting L-PBF for manufacturing the knee resection inserts included in this work. This process uses the energy of a focused laser beam (typically Nd-YAG) to locally melt metal powder into a solid part [30]. An EOSINT M270 machine has been chosen to manufacture test specimens. This machine has a 250 × 250 mm working area, whereas building height could reach up to 215 mm. A 200 W Yb-Fiber is used alongside with F-Theta precision lens. Layer thickness could range from 20 to 100 µm, whereas other process parameters (scanning speed or effective power consumption) are material-dependent and established according to the indications of the manufacturer. Parts have been manufactured at PRODINTEC Technological Center. The estimated batch size was established at 1000 units per year, and parts must be manufactured according to the design shown in Figure 7. Tolerances were defined by the research team taking into account part functionality and meaningfulness regarding the objectives of this particular case study, but they are not intended to be applicable to other resection guide designs, since present design values for geometric tolerances values could possibly be too restrictive. 

This part includes two FoS affected by tolerances: external width (nominal value 4.5 mm with a symmetric tolerance of ±0.05 mm) and slot width (nominal value 1.35 mm with an asymmetric tolerance of +0 to +0.05 mm). Internal surfaces of the slot are also affected by a 0.15 mm flatness tolerance, whereas parallelism between those surfaces and the external ones has to be under 0.15 mm. Other part features do not demand specific tolerances, so it can be assumed that usual manufacturing quality would be adequate, since non-compliance of general tolerances related to these features would not critically affect instrument guiding during surgery. Verification will be carried out using a DEA Global Image 09-15-08 Coordinate Measurement Machine (CMM). This machine has been calibrated according to EN 10360-2:2001, with a maximum permissible error in length measurement (MPE_E_) of 2.2 + 3·*L*/1000 µm (*L* in mm) and a maximum permissible error in probing repeatability (MPE_P_) of 2.2 µm. PC-DMIS metrology software was used to perform verification operations. Temperature in the laboratory during verification procedures is maintained within 20 ± 2 °C. Once information has been gathered and the main features of the problem have been stated, the sequence of tasks continues with the definition of the Operation and Verification Spaces and the subsequent Standard Quality Assessment.

#### 3.1.1. Operation Space 

Influence Factors should be grouped in two categories: controllable and non-controllable (Table 1).

Since L-PBF has been selected as the most appropriate AM process for this work, and production shall be carried out in an EOS M270, neither process type nor machine could be initially considered as controllable factors. These decisions would also condition subsequent ones, since other possible influence factors, like the type of material, should be accordingly limited to those that the process/machine combination can handle. Therefore, since EOSINT M270 uses proprietary materials, possible materials are reduced to stainless steel PH1 or titanium Ti64. Since weight is not a crucial parameter, SS PH1 has been selected as the construction material, and therefore should be considered as a non-controllable factor. Geometry and dimensions have been set at the design stage. Consequently, all features, geometries and dimensions in the CAD have been also considered as non-controllable factors. Exceptions to this rule are those FoS affected by tolerances since, even when not subjected to modifications at this stage, they could be modified during the Design Optimization stage. Layer thickness in this machine ranges from 20 to 100 µm, so it is a controllable factor. Nevertheless, once layer thickness has been selected, volume rate, scanning speed and effective power are also defined according to material technology specifications. This implies that they cannot be modified by the operator and, consequently, they must be considered as non-controllable factors. This is also applicable to ambient parameters like nitrogen atmosphere (1.5% oxygen), building platform model, or base temperature (40°). Part orientation has a great influence upon processing time, which usually implies that parts are oriented so that minimum Z travelling is required. Nevertheless, issues regarding manufacturing of slot surfaces were taken into account to avoid excessive overhanging, which would require support structures inside the slot. This concern involved selecting a vertical orientation for the slot and, consequently, orientation has been labelled as a non-controllable factor. 

On the other hand, the location of parts within the workspace can be modified by the operator with minimal restrictions (like minimum allowed space between adjacent parts), so it should be considered as a controllable factor. Support structure type is also a controllable factor. Finally, post processing operations could also have an influence upon quality. A support removal operation is unavoidable if support structures are used. Other post-processing operations, like sand blasting or thermal treatments, are optional, and so they should be considered as controllable factors.

#### 3.1.2. Verification Space 

QI would be related to part FoS, affected by dimensional and geometric tolerances. They would also be ranked according to their relevance to part functionality. 

Accordingly, the distance between parallel surfaces of the slot (*DS*) has been considered as the most relevant QI, since it critically affects resection instrument performance during surgery. An excessive distance would cause noticeable clearance between the slot and the resection instrument, whereas an insufficient distance would make its movement difficult. The second most relevant, Flatness of Slot parallel surfaces (*FSR* for the Rear surface and *FSF* for the Frontal surface) could also have an influence during resection, while Parallelism between these Slot surfaces (*PS*) must be controlled in order to allow uniform behavior of the instrument with independence in its orientation during resection. Finally, Distance between External surfaces (*DE*) has a relatively lower relevance, since its insertion in the alignment part would be favored by PA flexibility. QR have been defined as the acceptable range of values that each QI should adopt. These limits have been established during the design stage, as reflected in Table 2.

#### 3.1.3. Standard Quality Assessment 

Standard Quality Assessment implies manufacturing a test set and checking if the QI values measured during verification fulfil QR. In order to manufacture the test set, all controllable factors must be revised, and a Standard Process Configuration defined. This task should be done by taking into account existent know-how, which should include the literature or research works, supplier recommendations or personnel’s previous experience. Consequently, layer thickness was set at 20 µm, according to the recommended value for SS PH1. Layer thickness selection determines other variables that, like Volume Rate, fixed at 1.8 mm^3^/s, are included in the technology files provided by the manufacturer. Part location could also affect quality but, since the objective is to manufacture medium-to-high batches, it is necessary to accommodate the maximum possible number of units in each tray. Accordingly, it was decided that parts within the test set would be distributed along the whole work area. A lightweight supporting structure was selected, since it is the easiest to remove and minimizes removal cost. The same criterion was used to decide that no thermal processing would be applied for the standard configuration. On the other hand, since sand blasting is used to minimize the effect of metallic projections upon surface quality, this post-processing operation was included as part the process. Once the values and alternatives for all these factors have been defined, Process Configuration could be considered complete. 

**Figure 8:**
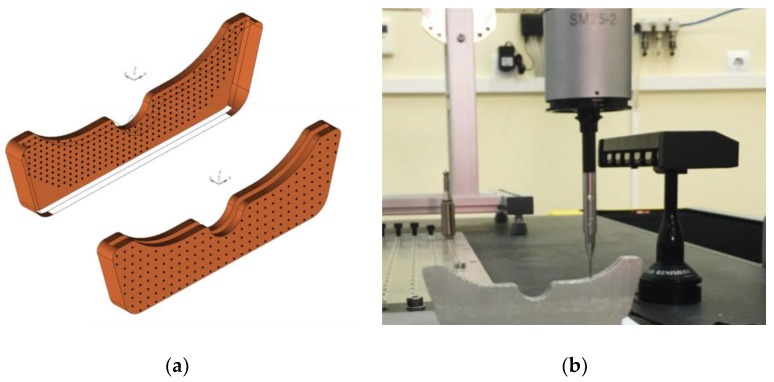
Verification Procedure. (**a**) Distribution of points on the internal and external surfaces; (**b**) CMM measurement of a test specimen.

Regarding the Verification Procedure, it has to be noted that QI could be calculated using just four planes adjusted to slot parallel surfaces and external parallel surfaces. To digitize each internal surface, a regular grid with 284 points was used. The distance between adjacent points is 1.4 mm in the same column and 1.6 mm in the same row. Due to slot restricted accessibility, a spherical-end stylus probe with 0.7 mm diameter and 20 mm length was used. In the case of external surfaces, regular grids with 171 digitized points were used. The distance between adjacent points was 2.2 mm in the same column and 2.26 mm in the same row. Complete digitizing of each part in this work (including alignment routine) was repeated thrice, and, each time, four planes were adjusted to each set of digitized points (Figure 8). Consequently, QI was calculated thrice, and average values were obtained. 

The test set used to check part quality under standard process configuration was arranged as a series of sixteen test specimens, manufactured in four independent trays (four units each). These parts were located on the corners of the tray, so that the effect of part location upon QI could be observed through results’ analysis. Once the specimens were manufactured, parts were removed by mechanical means and sand blasted before CMM verification at the laboratory. Table 3 collects the average values of the three measurements performed for each QI and each part.

Results clearly indicate that QR would not be fulfilled under standard manufacturing conditions. DS average value (1.413 mm) indicates that slots tend to be wider than expected (13 µm wider than the correspondent QR upper limit). Additionally, DS standard deviation (0.040 mm) indicates an unexpectedly high variability. This indicates that the process is not under control and standard configuration would not allow for batch production. Similar conclusions can be derived from the analysis of the other QR, since none of them are fulfilled under standard conditions. Geometric indicators (PD, FSD and FSF) clearly exceed the desired limits, while simultaneously presenting very high values for the standard deviation. Finally, DE achieves the desired quality in nine out of sixteen parts. Nevertheless, DE standard deviation (0.060 mm) clearly indicates that an unacceptable percentage of parts would not fulfil this condition during batch manufacturing.

Consequently, Standard Quality Assessment reveals that QR are far from being fulfilled. This means that the methodology should move onto the second step: Process Optimization.

### 3.2. Step 2: Process Optimization

The objective of Process Optimization is to work exclusively upon process configuration parameters to fulfil quality requirements. This means that the level of significance that variations in controllable factors exert upon variation in QI values must be established. At this stage, controllable factors have been reduced to Layer Thickness, Part Location, Type of Support, Thermal Treatment and Sand Blasting. A DOE considering five parameters could be performed at this point to gain a statistical assessment of each factor’s relative influence upon QI. Nevertheless, an analysis of factor influence likeness has been previously performed, to evaluate if the number of factors could be reduced in the first iteration of Process Optimization

- Layer Thickness value was set during the Standard Quality Assessment at the minimum achievable level (20 µm). Delgado et al. [31] have not found a statistically significant effect of Layer Thickness upon dimensional error in L-PBF for test specimens with non-sloped surfaces (like the one analyzed in the present work). On the other hand, Nguyen et al. [32] reported an increase in dimensional accuracy with decreasing layer thickness, and this result seems to have been confirmed by Maamoun et al. [33]. Although it is sometimes difficult to compare results, no research has been found supporting the hypothesis that thicker layers could improve dimensional quality. Accordingly, since the minimum achievable value for Layer Thickness has already been used for the Standard Process Configuration, this factor should not be included during the Process Optimization step;
- Sand blasting is the usual finishing process in L-PBF, since it is intended to remove metallic projections that appear as spikes on part surfaces. Consequently, sand blasting is presumed to reduce the unevenness of surfaces. This means that suppressing sand blasting would probably cause an increment in flatness and parallelism deviations and also could affect dimensional quality. These arguments led to the consideration of sand blasting as an unavoidable step;
- Part location within the working area could influence quality results, and this hypothesis could be tested using data from the previous step. Analyzing how DS measures are related to part location within the trays, the Pearson Correlation provides a p-value of 0.721. This value reveals an extremely low probability of a linear relationship between DS and part location for the standard process configuration. Additionally, none of the other indicators (FSR, FSF, PS and DE) show any correlation with part location according to their p-values. This means that variability in QI cannot be explained by part location within the tray, and, thus, modifying location would not allow for fulfilment of QR;
- Lightweight supporting structures were preferred during the Standard Quality Assessment because part removal was easier and did not demand subsequent machining. Nevertheless, manual removal of support could have an influence upon observed lack of quality [34], so Type of Support should be included as a possible influence factor in the Process Optimization step;
- Similarly, thermal stresses accumulated during manufacturing operations (in-layer and layer-upon-layer) could provoke a noticeable distortion in the part, after they are released from support [35]. Accordingly, thermal treatment should be included in the Process Optimization step.

**Figure 9:**
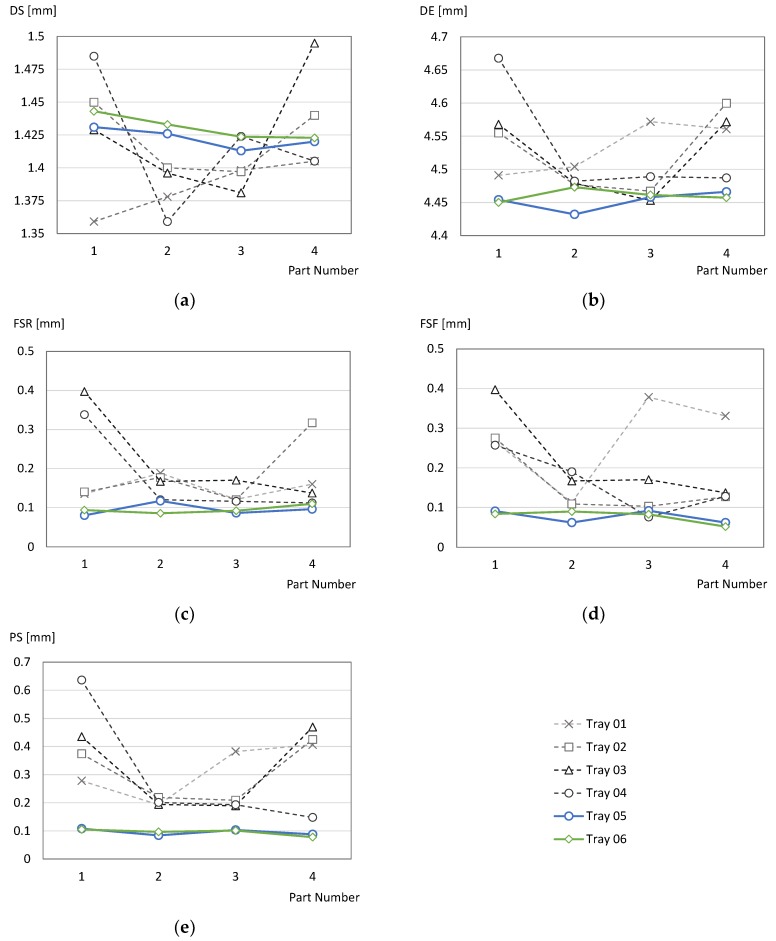
Comparison of QI measures for trays 01 to 06: (**a**) *DS*; (**b**) *DE*; (**c**) *FSR*; (**d**) *FSF*; (**e**) *PS*.

As a result of the analysis, only two factors were left for Process Optimization: Type of Support and Thermal Treatment. In order to check if those factors have a real influence upon Quality Indicators, it was decided that two additional trays (Tray 05 and Tray 06) should be manufactured using solid support (instead of a lightweight one) and applying a thermal treatment before releasing parts from the tray. Thermal treatment was intended to release thermal residual stresses of parts and was carried out following the recommendations of the material supplier (EOS) and manufacturer (PRODINTEC), by maintaining the tray at a 482 °C during four hours in a Nabertherm oven. Then, the parts and tray were left to cool at room temperature before being taken to a sawing operation. Once each part was released from the tray, it was taken to a Computer Numerical Control (CNC) milling machine to complete support removal. An *ad hoc* designed jig was used to prevent part deformation during milling. When the overall process had finished, parts were verified with the CMM using the same procedure as for trays 01 to 04. Comparisons between values of QI calculated from trays 01 to 04 (lightweight support/no thermal treatment) and 05 to 06 (solid support/thermal treatment) are provided in Figure 9.

Results indicate that the variability observed during the first step was related to the factors included in this Process Optimization step, since the QI measured for parts from trays 05 and 06 are clearly more uniform. This result is especially remarkable in the case of QI derived from geometric tolerances, since all the parts in the new trays fulfil QR for *FSR*, *FSF* and *PS*.

In the case of dimensional requirements, none of the new parts fulfil QR for *DS*, whereas only six parts fulfil the requirement for *DE* (although *DE* values are particularly close to the lower acceptable value). Nevertheless, the most important fact about measured dimensions is that variability seems to be significantly lower than that observed during the first step. Standard deviation results for dimensional QI pointed to the possibility of fulfilling QR by means of an optimization of design parameters. 

Table 4 provides the measurement results of these eight specimens. The conclusion of this analysis is that the resection guide must be manufactured using solid support structures and that a thermal treatment, like the one described above, must be applied, and both elements have been thereafter incorporated to the Optimized Process Configuration. Manufacturing using this configuration directly allows the fulfilment of geometrical QRs, but it is still clearly unsuccessful in the case of the dimension of the slot and external width. 

Reaching this point, no additional QI improvement could be reasonably achieved by means of process parameters without acting upon design. Consequently, the methodology moved to the third step: Design Optimization.

### 3.3. Step 3: Design Optimization

Design Optimization can affect both dimensional and geometric tolerances, and the level of complexity required depends on the results observed during the previous stages. In this case study, geometric QR have been fulfilled via Process Optimization, so Design Optimization must focus on dimensional QR. Dimensional optimization implies building a mathematical model capable of accurately predicting the value that each QI would reach, as a function of those controllable factors whose significance has been considered relevant within the scope of the problem. In this case, study of both *DS* and *DE* present low variability after the Process Optimization step (*DS* standard deviation is 9 µm and *DE* standard deviation is 12 µm). This suggests that a unique linear compensation of designed theoretical values for all the parts within a tray could be applied. In the simplest formulation, the average deviation of both QI with respect to correspondent theoretical dimensions could be calculated and the design parameters modified accordingly, assuming linear behavior of results. 

Nevertheless, although this could be the optimal approach for *DE*, it would not be equally recommended for *DS*. Quality Requirement for *DE* has a 100 µm range; consequently, uniform compensation should reasonably get most of the parts within QR. However, the Quality Requirement for *DS* has a 50 µm range. In order to achieve further improvements of *DS*, it was decided that the level of significance of remaining controllable factors (part location on the tray, with respect to *X* and *Y* axis) upon *DS* measures variability has to be verified. Although these factors have not shown significance for parts manufactured under Standard Process Configuration, the reduction in variability achieved via Optimized Process Configuration could have modified this circumstance. 

Accordingly, a two-level full-factorial 2^2^ DOE has been defined. Possible curvature effects have been taken into account by including two central points. This design allows for using data from trays 05 and 06. Part location has been coded according to a virtual *XY* origin ideally placed at the geometric center of the tray. 

Parts located on the left side of the tray have been coded as *X* = −1, whereas those on the right side have been coded as *X* = 1. Similarly, parts located closer to the door have been coded as *Y* = −1, whereas those far from the door have been coded as *Y* = 1. Central locations have been coded *X* = 0 and *Y* = 0. Table 5 contains the structure of experiments for the DOE and the measured values for QI.

*DS* results (Table 5) have been processed using Minitab 17 statistical software to obtain variance analysis. Results are reflected in Table 6.

**Figure 10:**
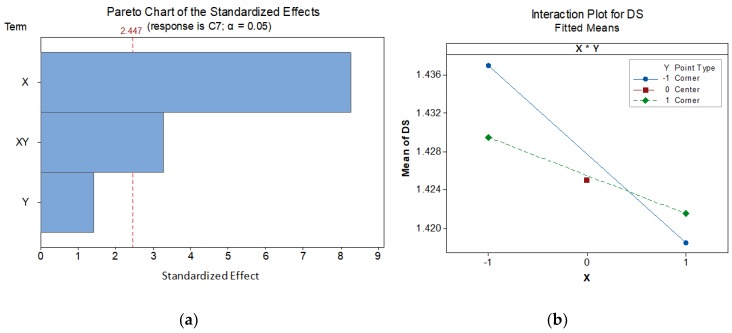
*DS* statistical graphs: (**a**) Pareto chart of the standardized effects; (**b**) Interaction plot.

Analysis of variance points out that the location along the *X* axis has a significant influence upon *DS* values. Additionally, although the *Y* location appears non-significant, the interaction of *X* and *Y* has also been found to be significant. This means that variance in *DS* could be modelled by considering the location of parts within the manufacturing tray, with respect to both *X* and *Y* axes, since a linear relationship between them could not be discarded. Figure 10 contains an explicative Pareto chart of standardized effects, where the relative significance of factors *A* (*X*), *B* (*Y*) and interaction A*B (X*Y) can be observed. Additionally, the interaction plot for *DS* helps to explain the effect of each factor upon *DS*. Parts located to the left tend to have a wider slot than parts located to the right. Parts located closer to machine door are expected to have wider slots than parts located far from the door when the left side of the tray is analyzed, but this behavior is flipped (wider slots for distant parts) when parts manufactured on the right area of the tray are analyzed.

This behavior illustrates the significance of X*Y interaction, and indicates that modelling *DS* variability would have to include both *X* and *Y* locations as parameters.

**Figure 11:**
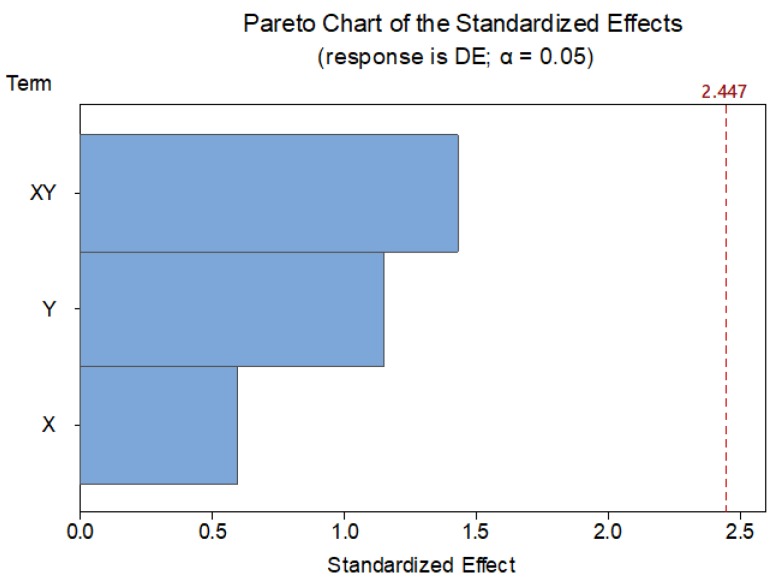
Pareto chart of the standardized effects for *DE*.

Additionally, values for center points indicate that a linear relationship between location and DS should be expected, since there is no evidence of curvature. Although there was no need to analyze X*Y significance upon *DE*, this task could be carried out without additional experiments. Consequently, an additional analysis of variance has been performed for *DE* using data from Table 5. Results indicate that neither *X* nor *Y* have any significance on DE variability. This result implies that observed variability cannot be explained by means of part location within tray, so these factors should not be taken into account when building a predictive model for DE. Figure 11 provides a Pareto chart for *DE*.

In sum, although the part location according to *X* and *Y* axes should be taken into account when predicting *DS* variability (values would differ significantly for different locations), they should not be considered for *DE* (values would be similar, independent of part location). Accordingly, no predictive model is required for *DE*. Instead, a simple linear compensation of the average values of correspondent QI will be used in the present case study.

#### 3.3.1. Predictive and Inverse Models 

To elaborate the predictive model for DS, some considerations must be given:

- Design values for *DS* will be thereafter denoted as DS_D_, whereas the correspondent values measured after part manufacturing will be denoted DS_M_. Equivalent notation will be used for *DE_D_* and *DE_M_*;
- Modifying *DS_D_* could not only affect *DS_M_*, but also *DE_M_* and, vice versa, modifying *DE_D_* could have and influence upon *DS_M_*;
- Predictive model should include design factor (*DS_D_*) as part of its parameterization. To achieve better results, the set of data that would be used to adjust such models should also include variations in design factors.

Cross-influence of *DS_D_* and *DE_D_* has been addressed in this case study by focusing on the predictive model for *DS_M_*, and calculating *DE_D_* compensation from average values of *DE_M_* obtained from those parts used to construct the *DS_M_* model. Since the distribution of *DS_M_* with part location have previously shown no curvature, a simple polynomial expression has been used for predictive model $DS_{M}=f_{1}\left(X,Y,DS_{D}\right)$ Consequently, *DS_M_* values should be predicted as a function of *X* location, *Y* location and *DS_D_*, being *a_1_*, *a_2_*, *a_3_* and *a_4_* coefficients that minimize the adjustment error of such a function (1).

(1) $$DS_{M}=a_{1}+a_{2}\times X+a_{3}\times Y+a_{4}\times DS_{D}$$

To calculate these coefficients, a new tray arrangement was defined so that the theoretical distance between slot parallel surfaces could also be taken into account. Consequently, two additional trays were defined and manufactured: the first one includes six parts, four located at the corners of the tray and the other two at the center, with a nominal *DE* of 1.350 mm. The second one follows the same distribution, but the width of the slot has been reduced to 1.250 mm. These limits have been selected according to previous results, which show that *DE_M_* tends to be noticeably higher than *DE_D_*. Consequently, optimized values for this parameter could be reasonably expected to be smaller than the initial ones and probably within the 1.350 to 1.250 mm range. Parts were thereafter measured and results can be found in Table 7.

Model coefficients have been calculated by means of a least square iterative method and results are provided in Table 8.

Once the predictive model was defined, an inverse model was constructed. This inverse model should provide an optimized design value for *DS* (*DS_O_*), so that the correspondent measured value *DS_M_* of the manufactured part is as close as possible to the theoretical (optimal) value for *DS* (*DS_T_*). This objective could be achieved by means of an optimization problem (2). Note that, in the original design, *DS_T_* and *DS_D_* were equivalent, whereas, after optimization, each part would have a different *DS_O_*.

(2) $$\min error=\min{\left(DS_{M}-DS_{T}\right)}^{2}=\min{\left(f\left(X,Y,DS_{D}\right)-DS_{T}\right)}^{2}$$

Consequently, the value that minimizes such functions should be an optimized *DS_D_* (denoted as *DS_O_* for clarification purposes). This problem could be efficiently solved by means of an interior-point method, taking advantage of the easily calculable derivatives of the function. In the present work, MATLAB *fmincon* command has been used to determine the optimal design values for *DS*.

Finally, *DE* compensation value was calculated from *DE_M_* results obtained from Table 7 parts, so that the average value reflects possible variations derived from *DE_D_* modification. Accordingly, a *DE_M_* average value of −0.043 mm has been calculated and linear compensation should provide an optimized value of 4.532 for *DE_O_*.

#### 3.3.2. Optimized Design Quality Assessment 

To evaluate fulfilment of QR once the design was optimized, an inverse model was used to design a verification tray with nine parts. This tray included six positions that had already been used to elaborate the prediction model and four additional intermediate ones (never used before). According to the predictive and inverse models, the design dimensions of slots were different for every individual part, whereas *DE_O_* is unique (4.543 mm) for all parts. Table 9 includes design values and measured results. 

**Figure 12:**
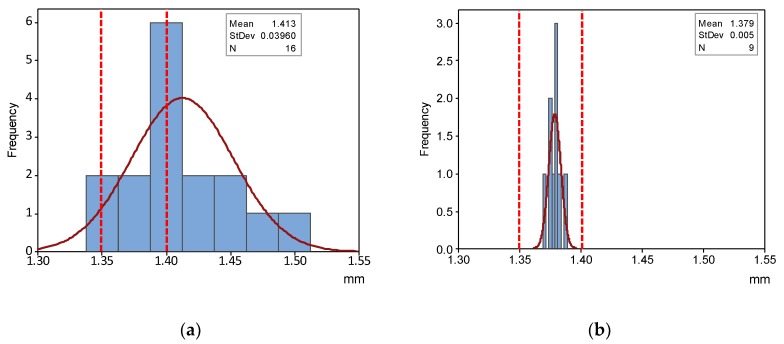
Histograms of *DS_M_* values: (**a**) Initial; (**b**) Optimized.

**Figure 13:**
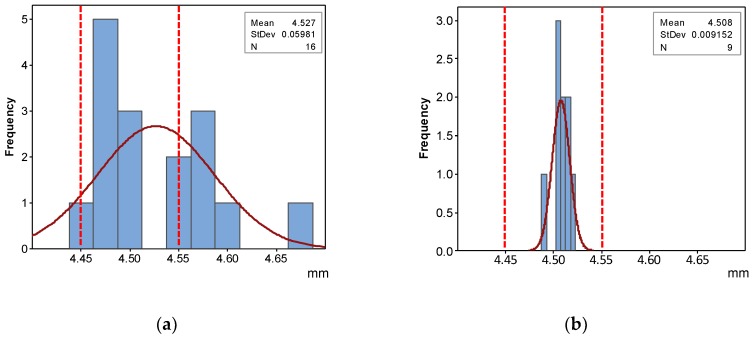
Histograms of *DE_M_* values: (**a**) Initial; (**b**) Optimized.

As can be observed, QR have been fulfilled for every manufactured part in both *DE* and *DS*. To illustrate the evolution of QI from the Standard Process Configuration to the last step of the optimization procedures, Figure 12 and Figure 13 provide two comparative histograms of *DS_M_* and *DE_M_*.

It can be observed that improvement was achieved by two effects: centering the average value of slot width with respect to the limits of the correspondent QR, and reducing initial variability to the extent that an extremely high percentage of parts should fulfil required quality. In fact, standard deviation within the verification tray has been reduced to only 5 µm, whereas its value calculated for the Optimized Process Quality Assessment was 9 µm. QR for DE has also been achieved via Design Optimization but, since the same dimensional compensation has been used, standard deviation presents a similar value (9 µm).

According to these results, batch production could commence once the implications of Process and Design optimization steps have been incorporated into production configuration.

## 4. Discussion

The proposed framework allows for combining the advantages of different approaches analyzed in the literature review. Some of the works related to the error analysis and error prevention [6,7,14] could be incorporated under our approach to the Process Optimization stage. In fact, once consolidated, their conclusions on factors’ influence upon part quality could be part of an intensive knowledge base to simplify decision-making procedures in AM. Similarly, the mathematical methods proposed for error prediction used in works devoted to error correction [17,18,22] could be easily incorporated into the Design Optimization stage. However, the definition of this framework makes it unnecessary to apply optimization to the whole part, but instead is focused on FoS affected by dimensional tolerances and their related geometrical tolerances. A global compensation of geometric distortions [22,23] could be researched, to see if it is preferable to our proposal of a specific compensation, but there is a risk of orientating efforts to models that, despite being able to improve overall dimensional and geometric quality, failed to fulfil a specific tolerance. Nevertheless, both approaches should not be considered exclusive, since it is possible to anticipate that, in the future, machine learning and artificial intelligence approaches [10,21] will both be incorporated to machine control systems. The proposed framework would check if specific QR have been fulfilled once optimization procedures have been incorporated into machine technology and, in case embedded optimization rules are still not enough to match tolerances, it would provide a methodology that manufacturers could easily follow. The degree of complexity of the tasks included in the proposed framework is highly dependent on each particular part’s design and process characteristics, but it encourages taking advantage of available knowledge to simplify experimental effort. Models capable of accurately predicting dimensional and geometric errors grow in complexity with the number of factors contemplated, so an approach that delays simulation efforts until problem complexity has been reduced (minimizing factors) will probably be more useful to end-use manufacturers in the short term. Further research should be done in order to provide detailed rules on how some decisions should be adopted. Quantitative evaluation of the possible consequences of opting for one process configuration alternative could help staff to reach higher levels of objectivity while minimizing subjective decisions. Moreover, defining a model that describes all available possibilities in terms of statistical analysis and mathematical modelling, while simultaneously helping staff to decide which of these tools should be preferable for a particular situation, would be of great use. 

## 5. Conclusions

The achieved results support the usefulness of a systematic framework for dimensional and geometric quality enhancement of additively manufactured parts. Work Analysis has permitted a reasonable understanding of the role of different influence factors, grouped according to controllable and non-controllable categories, to define both the Operating Space and the standard process configuration. An initial evaluation of QR fulfilment by means of a verification procedure and a test set provides an idea of the differences between measured QI values and QR objectives. Sorting controllable factors according to their relative influence upon QI values helps to simplify the Operational Space and drives testing to the most promising configurations. This contributes to a reduction in experimental effort and helps save costs and time. A balance between know-how and experimental effort should allow for an improvement in part quality that could eventually make further development unnecessary. Nevertheless, once the process’s capabilities have been exhausted, the possibility of working upon the design parameters, or even upon the parameterization model itself, could take part quality to a higher level. The application of this framework to a quasi-industrial case, involving dimensional and geometrical optimization of surgical-steel tibia resection guides, helps explain the proposed workflow in more detail. In fact, L-PBF manufacturing of these inserts has an approximate cost-per-unit of 37 €, according to PRODINTEC, with an approximate production rate of 1.6 units per hour (based on 50 specimen trays). This cost is truly competitive with the conventional manufacturing alternatives, ranging from 100 to 300 €. These results help to reinforce the idea that the proposed framework could contribute to the global objective of AM quality improvement.

## Figures and Tables

**Table 1:** Operation Space.

Category	Factor–Level 1	Factor–Level 2	Controllable	Non-Controllable
Production				
	Batch Size			X
	Type of process			X
	Equipment			X
Design				
	Geometry			X
	Dimensions			X
	Material			X
Process				
	Process Parameters			
		Layer Thickness	X	
		Volume Rate		X
		Scan Speed		X
		Power		X
		Ambient Parameters		X
		Base Temperature		X
		Part Orientation		X
		Part Location	X	
		Type of Support	X	
	Post-Processing			
		Support Removal		X
		Thermal/Heat Treatment	X	
		Sand Blasting	X	

**Table 2:** Quality indicators with their correspondent quality requirements sorted by relevance.

Priority Order	QI	Lower Limit (mm)	Upper Limit (mm)
1	*DS*	1.350	1.400
2	*FSR*	-	0.150
3	*FSF*	-	0.150
4	*PS*	-	0.150
5	*DE*	4.450	4.550

**Table 3:** Standard Quality Assessment results.

Item	Tray	Location	*DS* (mm)	*FSR* (mm)	*FSF* (mm)	*PS* (mm)	*DE* (mm)
1	01	1	1.359	0.135	0.266	0.278	
2	01	2	1.378	0.188	0.113	0.193	4.504
3	01	3	1.398	0.121	0.378	0.382	4.572
4	01	4	1.405	0.160	0.331	0.406	4.561
5	02	1	1.450	0.140	0.275	0.374	4.555
6	02	2	1.400	0.177	0.109	0.219	4.477
7	02	3	1.397	0.120	0.103	0.209	4.467
8	02	4	1.440	0.317	0.127	0.425	4.600
9	03	1	1.429	0.123	0.397	0.435	4.568
10	03	2	1.396	0.125	0.167	0.194	4.479
11	03	3	1.381	0.141	0.170	0.189	4.453
12	03	4	1.495	0.295	0.137	0.469	4.572
13	04	1	1.485	0.338	0.257	0.636	4.668
14	04	2	1.359	0.120	0.190	0.201	4.482
15	04	3	1.424	0.116	0.076	0.193	4.489
16	04	4	1.405	0.112	0.128	0.148	4.487
Average (mm)			1.413	0.171	0.201	0.309	4.527
Standard Deviation (mm)			0.040	0.076	0.103	0.140	0.060
QR Fulfilment			43.7%	62.2%	43.7%	6.25%	56.2%

**Table 4:** Optimized Quality Assessment results.

ID	*DS* (mm)	*FSR* (mm)	*FSF* (mm)	*PS* (mm)	*DE* (mm)
Average	1.427	0.095	0.077	0.096	4.456
Standard Deviation	0.009	0.013	0.016	0.011	0.012
QR Fulfilment	0%	100%	100%	100%	75%

**Table 5:** Design of experiments (DOE) structure and results.

ID	*X*	*Y*	*DS* (mm)	*FSR* (mm)	*FSF* (mm)	*PS* (mm)	*DE* (mm)
1	−1	−1	1.431	0.080	0.091	0.108	4.454
2	−1	1	1.426	0.117	0.062	0.084	4.432
3	0	0	1.421	0.078	0.057	0.070	4.458
4	0	0	1.423	0.089	0.082	0.089	4.453
5	1	−1	1.413	0.086	0.092	0.103	4.458
6	1	1	1.420	0.096	0.062	0.088	4.466
7	−1	−1	1.443	0.094	0.084	0.105	4.450
8	−1	1	1.433	0.085	0.090	0.097	4.473
9	0	0	1.428	0.088	0.077	0.080	4.452
10	0	0	1.428	0.092	0.083	0.101	4.455
11	1	−1	1.424	0.092	0.083	0.101	4.461
12	1	1	1.423	0.110	0.052	0.078	4.457

**Table 6:** *DS* Variance Analysis.

Source	DF	Adj SS	Adj MS	*f*-Value	*p*-Value
Model	5	0.000592	0.000118	23.11	0.001
Blocks	1	0.000169	0.000169	32.93	0.001
Linear	2	0.000361	0.000181	35.24	0.000
*X*	1	0.000351	0.000351	68.51	0.000
*Y*	1	0.00001	0.00001	1.98	0.209
2-Way Interactions	1	0.000055	0.000055	10.76	0.017
X*Y	1	0.000055	0.000055	10.76	0.017
Curvature	1	0.000007	0.000007	1.37	0.286
Error	6	0.000031	0.000005		
Lack-of-Fit	4	0.000029	0.000007	7.19	0.126
Pure Error	2	0.000002	0.000001		
Total	11	0.000623			
Model Summary					
S	R-sq	R-sq(adj)	R-sq(pred)		
0.0022638	95.06%	90.95%	73.96%		

**Table 7:** *DS* values used for predictive model construction.

ID	*X* (mm)	*Y* (mm)	*DS_D_* (mm)	*DS_M_* (mm)
1	−77.25	−90.58	1.25	1.311
2	−77.25	90.58	1.25	1.307
3	−12.25	0	1.25	1.329
4	12.25	0	1.25	1.331
5	77.25	−90.58	1.25	1.319
6	77.25	90.58	1.25	1.306
7	−77.25	−90.58	1.35	1.437
8	−77.25	90.58	1.35	1.430
9	−12.25	0	1.35	1.424
10	12.25	0	1.35	1.426
11	77.25	−90.58	1.35	1.418
12	77.25	90.58	1.35	1.421

**Table 8:** Predictive model coefficients.

Coefficient	Estimated Value
*a_1_*	−4.32500 × 10^−2^
*a_2_*	−3.25450 × 10^−5^
*a_3_*	−2.89799 × 10^−5^
*a_4_*	1.08833 × 10^−0^

**Table 9:** Optimized Design Quality Assessment results.

ID	*X* (mm)	*Y* (mm)	*DS_O_* (mm)	*DS_M_* (mm)	*DE_O_* (mm)	*DE_M_* (mm)
1	−77.25	−90.58	1.298	1.374	4.543	4.507
2	−77.25	90.58	1.303	1.378	4.543	4.508
3	−38.625	−45.29	1.301	1.383	4.543	4.506
4	−38.625	45.29	1.303	1.380	4.543	4.503
5	0	0	1.303	1.371	4.543	4.488
6	38.625	−45.29	1.303	1.374	4.543	4.508
7	38.625	45.29	1.305	1.380	4.543	4.514
8	77.25	−90.58	1.303	1.381	4.543	4.514
9	77.25	90.58	1.308	1.387	4.543	4.521
